# Aging-related changes in neuromuscular control strategies and their influence on postural stability

**DOI:** 10.1038/s41598-025-15444-4

**Published:** 2025-08-17

**Authors:** Scott J. Mongold, Christian Georgiev, Thomas Legrand, Esranur Yildiran Carlak, Antonella Iannotta, Pierre Cabaraux, Gilles Naeije, Marc Vander Ghinst, Mathieu Bourguignon

**Affiliations:** 1 Laboratory of Functional Anatomy, Université libre de Bruxelles (ULB), 1070 Brussels, Belgium; 2 Laboratoire de Neuroanatomie et Neuroimagerie translationnelles, UNI – ULB Neuroscience Institute, Université libre de Bruxelles (ULB), Brussels, Belgium; 3 School of Electrical and Electronic Engineering, University College Dublin (UCD), Dublin, Ireland; 4 Department of Neurology, Neurorehabilitation Ward, Hôpital Universitaire de Bruxelles (HUB), Université libre de Bruxelles (ULB), Brussels, Belgium; 5 Centre de Référence Neuromusculaire, Department of Neurology, CUB Hôpital Erasme, Université libre de Bruxelles (ULB), Brussels, Belgium; 6 Service d’ORL et de Chirurgie Cervico-Faciale, CUB Hôpital Erasme, Université libre de Bruxelles (ULB), Brussels, Belgium; 7 WEL Research Institute, Avenue Pasteur, 6, 1300 Wavre, Belgique

**Keywords:** Motor control, Musculoskeletal system, Geriatrics

## Abstract

Altered neuromuscular strategies are suggested to contribute to age-related decreases in postural stability. Current approaches tend to overlook global (whole body) neuromuscular postural control strategies, potentially due to methodological constraints or residual influence from a longstanding, but outdated, biomechanical view in which postural sway is represented by a single-jointed inverted pendulum. In this study, we investigate age-related differences in postural strategies during upright static balance maintenance by assessing global neuromuscular control. We collected simultaneous posturography and electromyography (EMG) data from young (18–35 years, n = 32) and older (65–85 years, n = 33) participants while they stood upright on a force plate or on foam pads thereon, with eyes open or closed. Postural instability was assessed by the standard deviation and velocity of the center of pressure. EMG sensors recorded the activity of thirty muscles (15 on each hemibody). Co-contraction across all muscle pairs was measured with Falconer’s co-contraction index (CCI), and muscle synergy with non-negative matrix factorization. The older group possessed increased global co-contraction intensity, marked by more frequent use of a knee extensor synergy, and was more unstable than the younger group. Notably, advancing age modulated the variability of co-contraction intensity, where the oldest individuals consistently adopted a pure co-contraction strategy marked by the highest CCI values and lowest variability. Age-corrected correlations revealed that knee extensor CCI values were significantly related to postural instability. Taken together, global co-contraction appears to be a signature of elderly postural strategy and age-related instability may be directly related to the extent of knee extensor co-contraction. These results stress the importance of zooming out from classical agonist–antagonist muscle pair investigations in the endeavor to understand elderly postural control strategy.

## Introduction

Maintaining a stable posture is a prerequisite for almost all locomotive behaviors. However, as we age, this ability appears to be compromised, as has been reported across many studies showing that the elderly are less stable than their younger counterparts. This has been evidenced in studies of single-leg stance, in which community dwelling older people tend to have more frequent postural adjustments than younger people^1^, and in studies of bipedal stance where older people (> 56 years) have larger body sway displacements and increased velocities in both eyes-open and eyes-closed conditions, compared to younger participants^2^, hindering the ability to maintain their center of mass over their base of support^3^. In parallel, individuals over 65 years old are known to have an increased fall risk^4^. Crucially, previous work suggests that the aging nervous system plays a substantial role in altered postural control, evidenced by different control strategies, typically involving increased co-contraction of antagonist muscles^3,5–7^. Identifying the physiological signatures of such postural control strategies that may be indicative of instability is key to better understand and prevent falls in healthy older adults aged 65 and above.

Neuromuscular postural strategy is often assessed through the co-contraction index (CCI). This index measures the extent of simultaneous muscle contraction, as derived from electromyography (EMG) activity, among a muscle pair^8,9^. Most postural-related studies that use the CCI examine the relationship between dorsiflexor and plantar flexor muscle activity and have shown increased co-contraction in aging^9–11^. However, age-related increases in muscle co-contraction appear to extend to other muscle pairs across the body, such as in the trunk during walking^12^, between knee extensors and flexors during stair-related activities^13^, and in the upper limb during cycling^14^. Whether this pattern persists across postural tasks with sensory perturbation or is evident in more proximal muscles in the leg, trunk, or the upper extremities is unclear. Assessing CCIs within a large set of muscles across the whole body may highlight other anatomical regions not often considered in postural control, potentially enabling the identification of unforeseen patterns of muscle co-contractions related to postural instability. Additionally, much of aging postural research broadly explores the effect of age by comparing younger to older participants^15–23^, leaving the nuanced evolution of postural decline in advancing age mostly unexplored. This is surprising given that aging is not a linear process and a clear description of the timeline of altered postural control would provide immense benefit for the implementation of targeted therapies.

While the CCI provides a quantitative method to describe muscle co-contractions, a higher-order method describing the organization of such co-contractions would further our understanding of neuromuscular control. Muscle synergy analysis is one such method^24–26^. Here, we use the term synergy to refer to weighted groups of muscles displaying a similar temporal profile of activation as identified mathematically from EMG data. In order to avoid the computational and energetic demands that would be required to coordinate the activities of skeletal muscles individually, it has been theorized that the central nervous system generates motor commands controlling a pre-organized combination of muscles^27,28^. In essence, muscle synergies are thought to streamline motor control, providing a translation between task level goals (e.g., stabilizing one’s center of mass) and execution level commands (activation of specific muscles) that are needed for task success^24,27^. While research exploring muscle synergies is vast and does include postural work^27–29^, age-related differences are unresolved.

The purpose of this study is to characterize differences in postural control strategy and stability as a function of aging, and determine how strategy may relate to stability. More specifically, this study aims to 1) identify differences in co-contraction patterns between young and elderly, 2) establish whether there are differences in the use of particular muscle synergies in aging, 3) within the elderly cohort, determine whether there is age-related modulation in co-contraction, and 4) determine whether co-contraction is related to the fluctuations of the center of pressure (COP), as assessed by posturography. In refining our understanding of age-related differences in postural control, we hope to provide potential diagnostic indices for the elderly and generate new targets for evidence-based interventions.

## Methods

### Participants

Sixty-five healthy participants took part in this study. Of them, 32 formed the young group (15 female; mean ± SD age, 25.5 ± 2.9 years; range 19–31 years) and 33 formed the older group (18 female; mean ± SD age, 71.9 ± 6.0 years; range 65–85 years). For some analyses, the older group was further separated into 3 old-age subgroups of 11 participants: young-old (7 female, 66.2 ± 0.6 years), middle-old (5 female, 70.7 ± 2.4 years), and old-old (6 female, 79.5 ± 3.3 years). Upon initial screening (pre-experiment), participants reported no history of neuromuscular disorders, orthopedic limitation, or balance-related disorders or medications affecting balance, and were self-reported as generally healthy. If participants responded to our screening indicating that they had any form of arthritis, they were excluded from participation. Participants were asked not to exercise within 48 h of the experiment and follow their typical sleep behavior. The experimental protocol was approved by the ethics committee of the Université libre de Bruxelles (CCB: B4062021000049, Reference: P2021/015). All participants gave written informed consent. The study was conducted in accordance with the Declaration of Helsinki. The sample size was consistent with, and in most cases larger than, those used in comparable studies of neuromuscular control of posture or gait, or those assessing age-related differences in COP parameters^9,11–13,16,19,20,23^.Table 1 presents participants’ demographics. Significant differences were found in average age and height, but not in weight or BMI.

**Table 1:** Demographics of young and older participants. Data are presented as mean ± SD.

Variables	Young group (n = 32)	Old group (n = 33)	*p*-value	*t*_*63*_
Age (years)	25.5 ± 2.9	71.9 ± 6.0	< 0.0001	− 39.5
Height (cm)	173.0 ± 10.0	165.9 ± 9.4	0.006	2.84
Weight (kg)	74.8 ± 14.6	71.9 ± 11.8	0.497	0.684
BMI (kg/m^2^)	25 ± 4.4	26 ± 3.9	0.220	− 1.24

### Experimental protocol

**Fig. 1:**
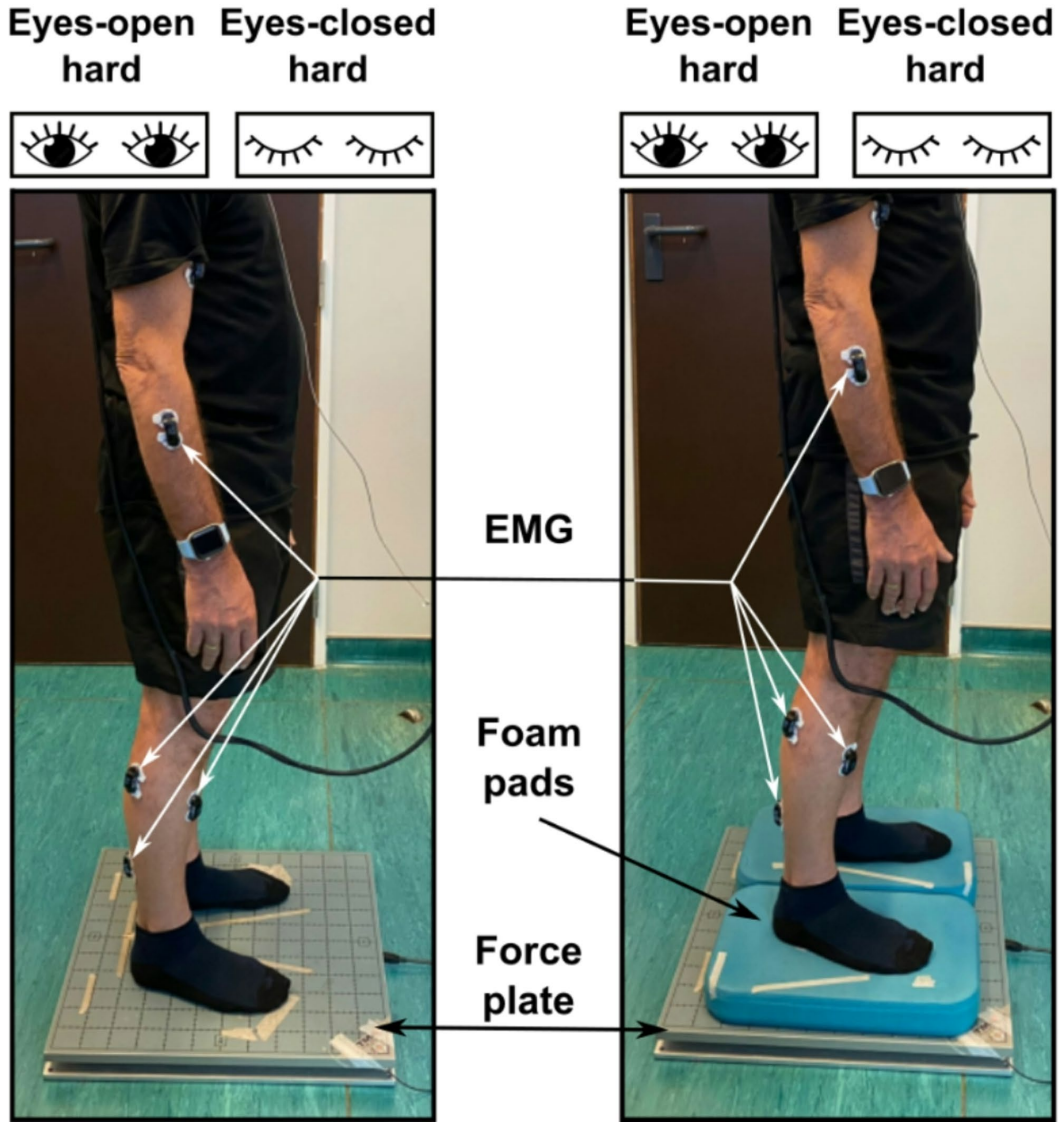
Experimental set-up. 15 EMG sensors on each hemibody, and stood on a force plate in 4 experimental conditions: either on a hard surface or on foam pads, and with eyes open or closed.

Figure 1 illustrates the experimental set-up. Photographed participant gave their written consent for the publication of their image in scientific journals. Participants completed 2 blocks of posturography, each consisting of 4 conditions performed in a randomized order. Each condition lasted for 5 min. Conditions consisted of standing on a force plate with eyes open (eyes-open hard) or eyes closed (eyes-closed hard), and on the force plate, but with foam pads under each foot (Domyos, Decathlon, Villeneuve-d’Ascq, France) with eyes open (eyes-open foam) or eyes closed (eyes-closed foam). Participants were allowed to wear socks (compression or specially textured socks were not allowed). Participants could not wear shoes for the experiment. In practice, over 80% of the participants wore socks. Participants were instructed to stand upright, stay relaxed, and maintain balance. Their feet were oriented in a comfortable position and spaced at approximately shoulder width. During a practice trial (30 s of standing), experimenters made sure to correct any tendency to weight-shift from one leg to the other. A red cross was placed approximately 1.5 m in front of the participant at eye level. This target served as a fixation point for each participant to encourage minimal movement of the head and shoulders over the course of each condition.

### Recording

**Fig. 2:**
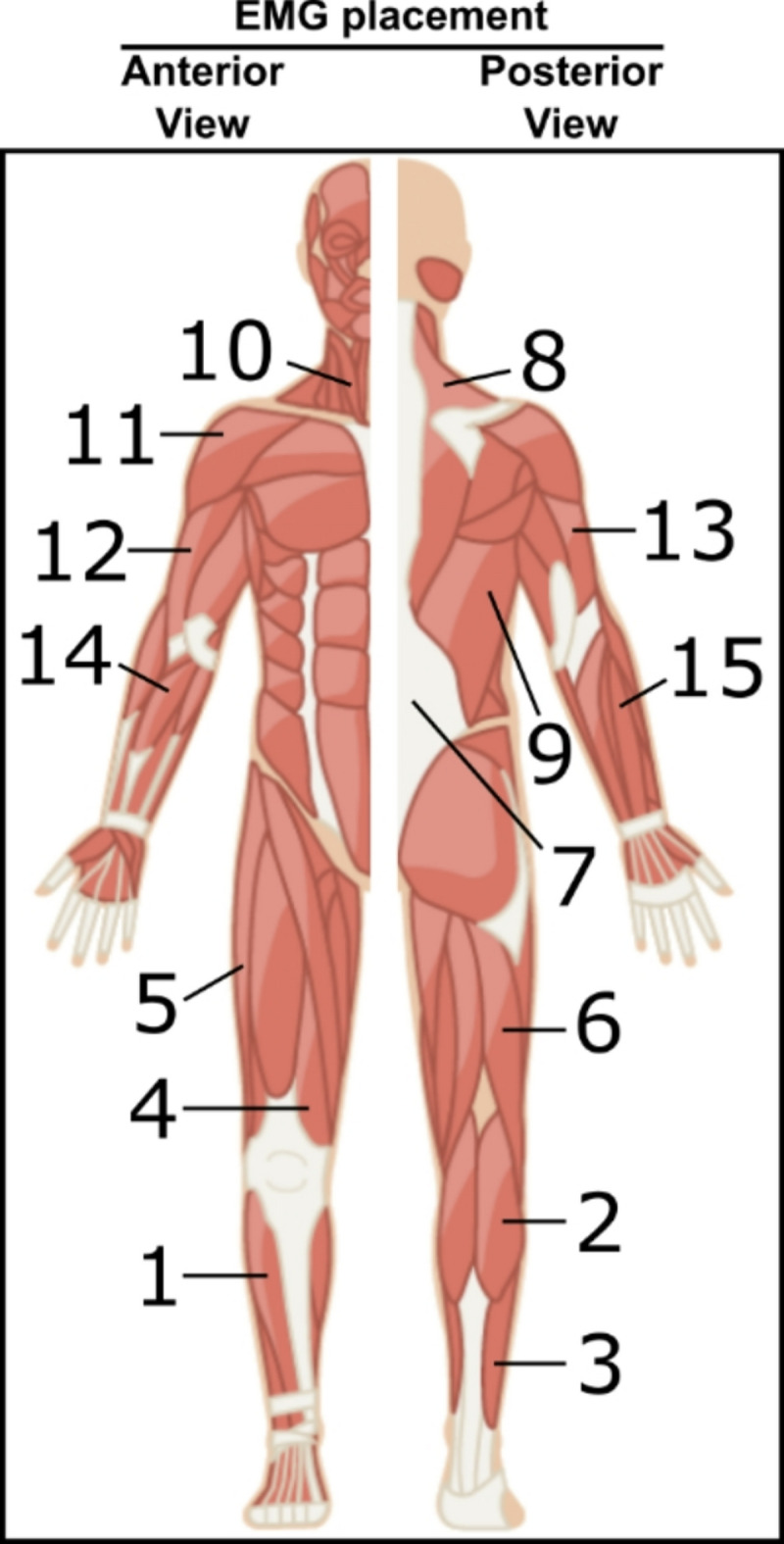
EMG placement. EMG electrodes were placed according to SENIAM guidelines (where applicable) over the TA (1), GL (2), SOL (3), VM (4), VL (5), BF (6), ES (7), TRAP (8), LD (9), SCM (10), MD (11), BB (12), TB (13), FDS (14), and EDS (15) of each hemibody. The image was adapted from iStock (credit to elenabs).

Participants were equipped with 30 bipolar EMG sensors (Pico EMG sensors, Cometa, Bareggio, Italy), 15 on each hemibody (see Fig. 2). Sensors were placed over the tibialis anterior (TA), gastrocnemius lateralis (GL), soleus (SOL), vastus medialis (VM), vastus lateralis (VL), biceps femoris (BF), erector spinae (ES), trapezius (TRA), middle deltoid (MD), biceps brachii (BB), lateral head of triceps brachii (TB), latissimus dorsi (LD), flexor digitorum superficialis (FDS), extensor digitorum superficialis (EDS), and the sternocleidomastoid (SCM), according to internationally-recognized guidelines, where applicable (SENIAM—www.seniam.org). Before recording, signal quality was confirmed by asking the participant to perform several concentric contractions of target muscles. EMG activity was recorded at 2 kHz during each condition. In addition, ground reaction forces and moments were recorded at 1 kHz with a force plate (AccuSway-O, AMTI, Watertown, MA, USA).

### Data processing

Force plate data was processed with custom scripts in Matlab (Mathworks, Natick, MA, USA). COP was calculated using the raw force and force moment data from the force plate. COP time-series were filtered between 0.2 and 10 Hz. Postural instability was quantified using two measures: i) the standard deviation of COP along the anterior–posterior axis (sdCOP_AP_) and ii) the mean absolute velocity of the COP along the anterior–posterior axis (vCOP_AP_). Increased sdCOP_AP_ and vCOP_AP_ denote sways with larger amplitude and speed, respectively. Both COP variables were normalized by participant height. The anterior–posterior direction was chosen in accordance with the selection of the lower extremity muscles, which control movement predominantly in the sagittal plane.

EMG data was processed with custom scripts in Matlab. First, data were low-pass filtered at 400 Hz and downsampled to 1 kHz. Following, EMG data and force plate data were synchronized, using a mechanical trigger before and after each 5 min standing condition. Heartbeat artifacts were removed from EMG data. For this, the timing of heart-beat artifacts (more precisely, of the peak of the R wave) was automatically identified, and used to build a template EMG response to heartbeat. This template, which encompassed the entire QRS complex, was regressed out from each EMG signal, at each timing of heartbeat. EMG data were then high-pass filtered at 20 Hz, rectified, low-pass filtered at 10 Hz, downsampled to 100 Hz, and normalized by their mean amplitude. As there are many EMG normalization approaches and there is currently no consensus on the optimal method for EMG normalization^30^, the chosen procedure was selected for its practicality and ease of implementation.

CCI values were computed following a previously reported formulation^8^. Unlike previous studies in which CCI was only computed for antagonistic pairs, we calculated CCI across all possible muscle pairs in successive 30 s windows. CCI values were then averaged across all windows.

For the muscle synergy analysis, only the lower extremity muscles and ES were included, similarly to previous studies^25,31^. The analysis was carried out by non-negative matrix factorization, as has been done previously^25,32–34^. Non-negative matrix factorization decomposed the time series of EMG amplitude arranged as a 2-D (channel × time) matrix into a specified number of synergies; each synergy being characterized by an activation time-course, which we did not analyze, and weights for muscles here called “motor modules.” Motor modules indicate combinations of muscles that tend to activate in synchrony. It has been shown that even complex human movements are simplified by a small number of motor modules, typically three or four^35^. Thus, the analysis was computed for 1, 2, 3, and 4 synergies. The variance accounted for (VAF) in EMG data was calculated for each synergy computation and further analyses were conducted with the number of synergies displaying a VAF of approximately 90% as done previously^36^. This threshold ensured that the selected number of muscle synergies could accurately reconstruct the original EMG signals^36^. A k-means clustering analysis was performed to identify and group motor modules across all participants and balance tasks. The ‘evalclusters’ function in Matlab was used to determine the optimal number of clusters according to the silhouette criterion, which were calculated for 1–15 clusters. The silhouette values range from –1 to 1. A high silhouette value indicates that data points are well matched to their own cluster, and poorly matched to other clusters.

### Statistical analysis

Statistical analyses were performed using Matlab and SPSS (v25, IBM, Armonk, NY, USA).

Between group differences in participant demographics were assessed using Independent samples t-tests.

A two-way ANOVA examined the effect of condition and age group on sdCOP_AP_, vCOP_AP_, VAF and on the global CCI values, which were estimated by averaging CCI values across all muscle pairs. Student t-tests with Bonferroni correction for multiple comparisons were used for post-hoc analyses. Effect size was calculated using partial eta-squared (η_p_^2^) with its 95% confidence interval (CI) also reported.

Permutation-based statistics were used to assess the significance of the difference in CCI between age groups. In this test, we first averaged the CCI values across participants within groups, and then estimated the difference in absolute value between the two groups, yielding a contrast of CCI value for each possible condition and muscle-pair. The significance of the mean of this value across conditions and muscle-pairs was assessed statistically. For this, its permutation distribution was built by estimating its value after having randomly assigned participants to the two groups. A p-value assessing the genuine difference in CCI values between groups was then assessed as the proportion of values in the permutation distribution being above the genuine value.

Permutation-based statistics were also used to assess the significance of local differences in CCI between age groups. More precisely, this test compared the value of the contrast between group-averaged CCI values for each muscle pair in each condition to their maximum statistics permutation distribution.

Permutation tests identical to those assessing CCI were used to assess the significance of the difference in frequency of use of the synergies between age groups. The measure quantifying this difference was the difference between groups in frequency of use of these synergies in absolute value. The global version of the test compared between groups this difference squared and averaged across all synergy clusters and conditions. The local version of the test compared between groups the maximum of this absolute difference across all synergy clusters and conditions.

Our CCI and synergy analyses revealed age-related differences in global CCI and use of a knee extensor synergy, where the VL and VM were co-contracting more frequently in the older participants. Therefore, subsequent analyses assessed global CCI and a knee extensor CCI to establish other age-specific changes in neuromuscular strategy. A lower leg-based (composed of TA, GL, SOL muscles) CCI was also used, as these muscles have been heavily characterized in balance-related literature^23,37–40^. The knee extensor and lower leg CCI values were obtained for each individual and condition by averaging the CCI values across all respective muscle pairs.

Two-way ANOVAs examined the effect of old-age subgroups (young-, middle-, and old-old) and condition on global, knee extensor, and lower-leg CCI values.

Driven by our results, an F-test was used to assess the equality of the variance in global, knee extensor and lower-leg CCI values between each pair of the three old-age subgroups.

A correlation analysis was used to estimate the strength of association between CCI values and pooled postural instability in the eyes-closed foam condition. Prior to that, outlier values deviating by more than ± 2.5 standard deviations from the mean were replaced with this threshold. This criterion was chosen to balance sensitivity to extreme values while minimizing unnecessary data loss. The same relationships were also assessed whilst controlling for age using partial correlation.

## Results

### Differences in age-related neuromuscular strategy

**Fig. 3:**
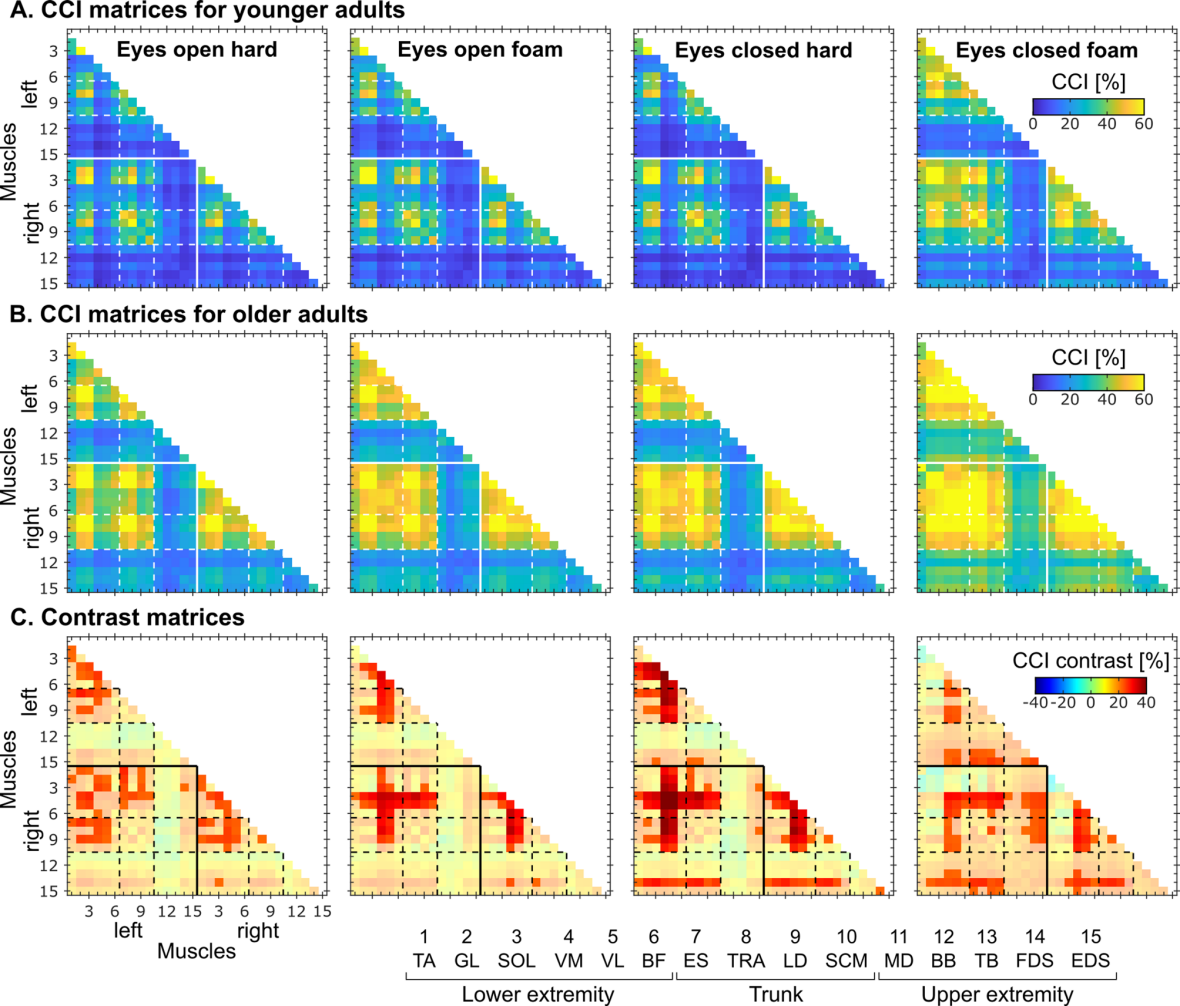
Muscle CCI matrices. (**A**) and (**B**)—CCI matrices for the younger (**A**) and older (**B**) participants in the 4 conditions. (**C**) Difference between CCI matrices for the older and younger groups. Brightened red values indicate significant increases in CCI values in the older group (*p* < 0.05, permutation-based statistics).

Figure 3 presents the CCI for all muscle pairs. Overall, CCI values appeared to be higher for muscle pairs among the lower extremity and trunk, to increase with condition complexity, and to be higher in older compared with young participants.

A two-way ANOVA revealed a significant effect of age (*F*_1,250_ = 212.9, *p* < 0.0001, η_p_^2^ = 0.46, 95% CI [0.37, 0.53]) and condition (*F*_3,250_ = 18.7, *p* < 0.0001, η_p_^2^ = 0.18, 95% CI [0.10, 0.26] ) on global CCI values, but no effect of their interaction (*F*_3,250_ = 0.27, *p* = 0.85, η_p_^2^ = 0.003, 95% CI [0.00, 0.02]). Post-hoc tests revealed that the older group had significantly increased global CCI values across conditions (*p* < 0.0001) and that global CCI values were significantly increased in the eyes-closed foam compared to the other conditions (*p* < 0.0001), with no significant difference between the other three (*p* > 0.48).

In examining the local CCI differences, the older group possessed significantly increased CCI across muscle pairs, both within and between hemibodies (*p* < 0.001; see Fig. 3, bottom row)*.* These increases in muscle pair co-contraction were especially visible for the knee extensor muscle pair (VM and VL) and muscle pairs formed of either the VM or VL and muscles of the lower extremity or trunk. For the conditions in which the eyes were closed, increases in co-contraction emerged between FDS or EDS with muscles of the lower extremity and trunk.

VAF increased from 1 synergy (mean ± SD, 60.6 ± 11.1) to 4 synergies (mean ± SD, 89.6 ± 3.6). Thus, further analyses were based on 4 synergies, similar to previous studies aiming to capture increased variance^26^. These synergies were then clustered into 8 groups, as determined by an average silhouette value of 0.61. Note that the same number of clusters was suggested by the Davies-Bouldin index (value = 0.98).

The ANOVA assessing VAF revealed a significant effect of age (*F*_1,250_ = 7.87, *p* = 0.005, η_p_^2^ = 0.031, 95% CI [0.003, 0.08]), but not of condition (*F*_3,250_ = 0.77, *p* = 0.52, η_p_^2^ = 0.009, 95% CI [0.00, 0.03]) and no interaction (*F*_3,250_ = 0.65, *p* = 0.58, η_p_^2^ = 0.008, 95% CI [0.00, 0.03]). The older group possessed increased VAF compared to the young (young, VAF = 88.9 ± 4.1, older, VAF = 90.2 ± 2.9).

**Fig. 4:**
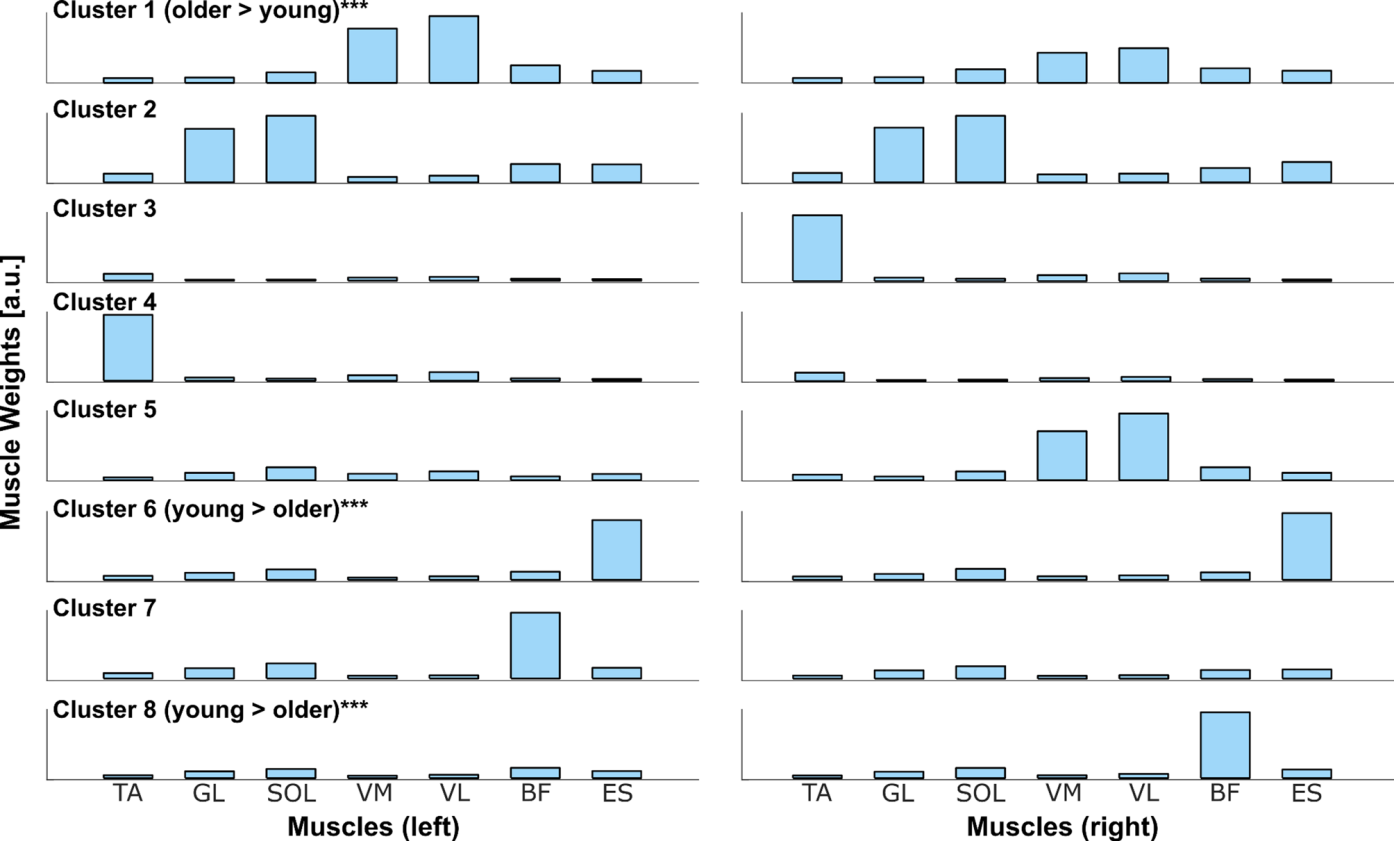
Motor modules for the 8 synergy clusters. ‘Older > young’ and ‘young > older’ indicate which group used the synergy cluster more often. ***, *p* < 0.001.

Figure 4 presents the 8 clusters of motor modules identified in the synergy analysis. The frequency of use of motor modules was different between the younger and older groups (*p* < 0.0001). Across conditions, 3 motor modules were used differently. Motor module cluster 1, which involved bilateral VL and VM (knee extensors), was more frequently used in the older group (*p* < 0.001). The motor module cluster 6, composed of bilateral ES, and motor module cluster 8, composed of right BF alone, were used more frequently in the younger group (*p* < 0.001). Other motor module clusters showed no difference in frequency of use between age groups (*p* > 0.05).

### Evolution of co-contraction in advancing age

**Fig. 5:**
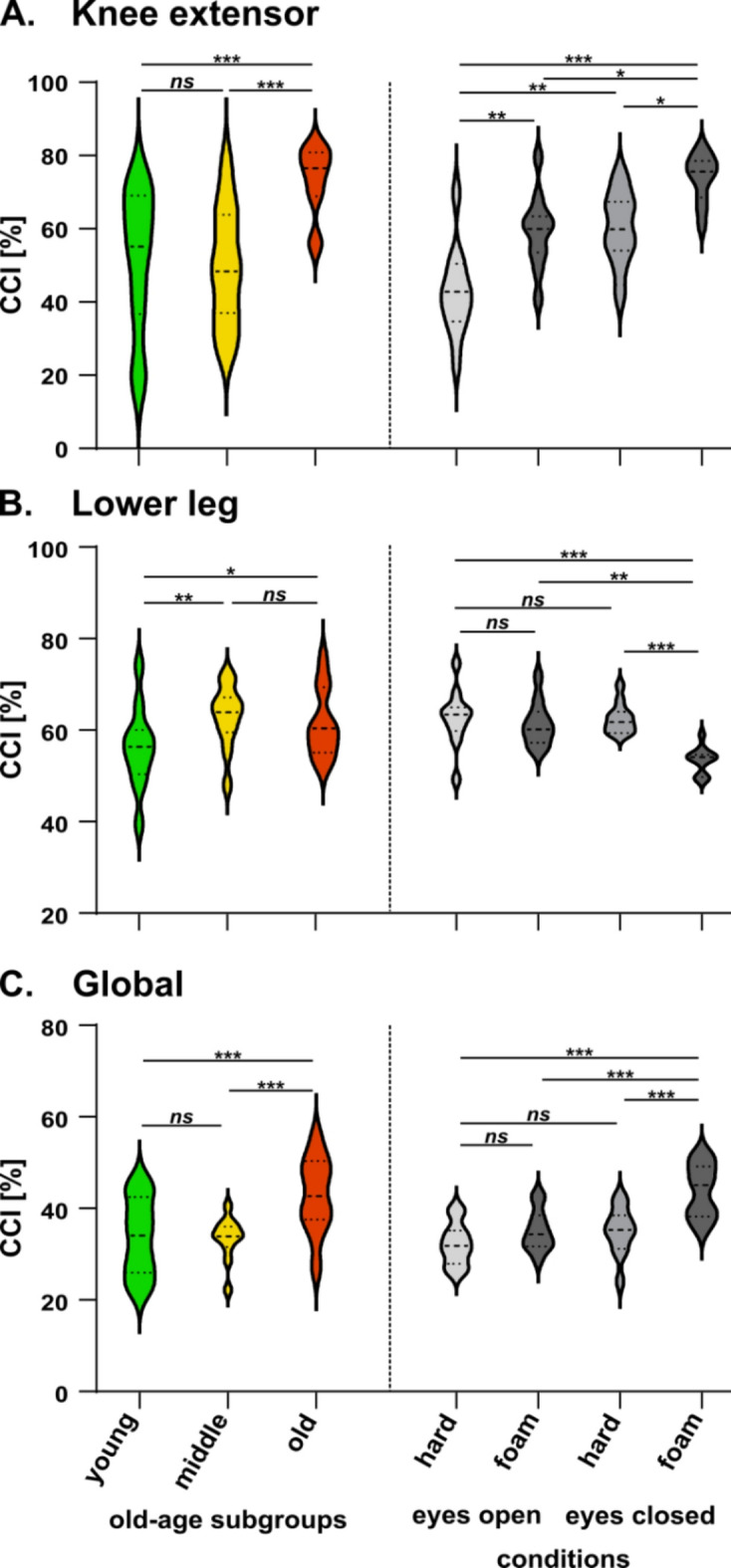
Distribution of older participants’ CCI values across old-age subgroups and conditions. Displayed are the knee extensor (**A**), lower leg (**B**) and global (**C**) CCI values. The darkened central line indicates the mean within each old-age subgroup or condition and dotted lines indicate quartiles. Significant differences between old-age subgroups and between conditions are indicated with horizontal lines (ns, not significant; *p < 0.05; **p < 0.001; ***p < 0.001).

Figure 5 presents the knee extensor, lower leg, and global CCI values for the 3 old-age subgroups and four conditions. The two-way ANOVAs assessing the effect of advancing age (old-age subgroups) and condition revealed a significant effect of advancing age (knee extensor CCI, *F*_2,119_ = 17.3, *p* < 0.0001, η_p_^2^ = 0.23, 95% CI [0.09, 0.32]; lower leg CCI, *F*_2,119_ = 6.67, *p* = 0.002, η_p_^2^ = 0.10, 95% CI [0.01, 0.19]; global CCI, *F*_2,119_ = 16.7, *p* < 0.0001, η_p_^2^ = 0.22, 95% CI [0.09, 0.32]) and condition (knee extensor CCI, *F*_3,119_ = 14.3, *p* < 0.0001, η_p_^2^ = 0.26, 95% CI [0.12, 0.36]; lower leg CCI, *F*_3,119_ = 8.51, *p* < 0.0001, η_p_^2^ = 0.18, 95% CI [0.05, 0.27]; global CCI, *F*_3,119_ = 10.9, *p* < 0.0001, η_p_^2^ = 0.22, 95% CI [0.08, 0.31]), and no significant interaction (knee extensor CCI, *F*_6,119_ = 1.69, *p* = 0.13, η_p_^2^ = 0.079, 95% CI [0.00, 0.14]; lower leg CCI, *F*_6,119_ = 0.82, *p* = 0.56, η_p_^2^ = 0.04, 95% CI [0.00, 0.07]; global CCI, *F*_6,119_ = 0.5, *p* = 0.81, η_p_^2^ = 0.02, 95% CI [0.00, 0.04]). Figure 5 presents the outcome of post-hoc comparisons.

Knee extensor and global CCI values were significantly higher in the old-old subgroup compared to the middle-old and young-old subgroups, with no significant difference between the two younger old-age subgroups. As for the lower leg CCI values, they were significantly higher in the middle-old and old-old subgroups compared to the young-old group, with no difference between the middle-old and old-old subgroups.

The knee extensor and global CCI values were significantly higher in the eyes-closed foam condition compared to the other conditions. In addition, knee extensor CCI values were higher in the eyes-closed hard and eyes-open foam conditions compared to the eyes-open hard condition. The lower leg CCI values were significantly lower in the eyes-closed foam condition compared to the other conditions.

**Fig. 6:**
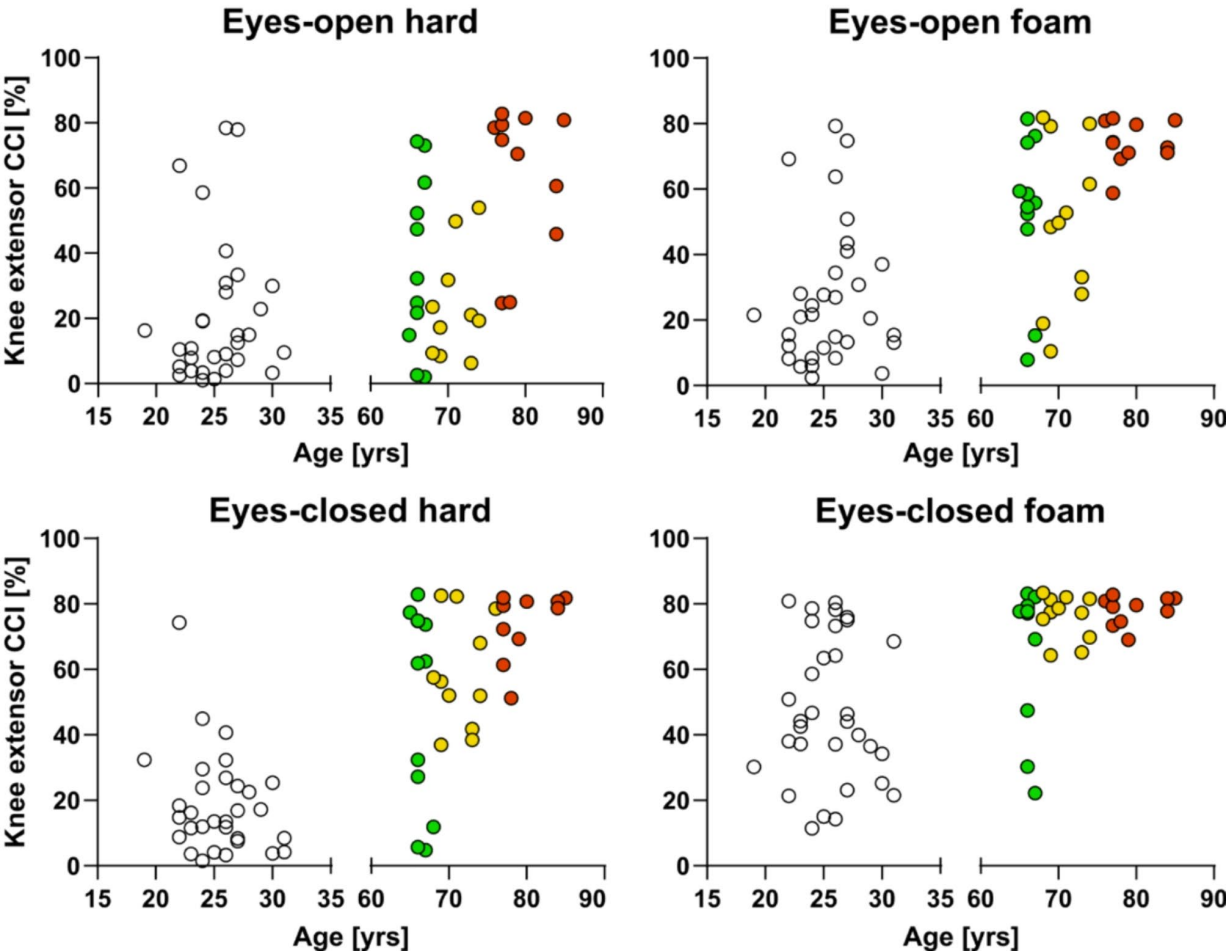
Relationship between age and knee extensor CCI across postural conditions. Circles indicate individual values and colors indicate old-age subgroups (green, young old-age; yellow, middle old-age; red, old old-age).

Figure 6 shows the relationship between knee extensor CCI values and age. It clearly highlights differences in variance between the old-age subgroups. Accordingly, a F-test identified significant differences in variance between the young-old and old-old subgroups in all conditions, except the eyes-open hard condition (*p* < 0.002), between the middle-old and young-old subgroups only in the eyes-closed foam condition (*p* = 0.009), and between the middle-old and old-old subgroups in the eyes-open foam and eyes-closed hard conditions (*p* < 0.05). Across all these comparisons, the younger subgroup displayed significantly higher variability.

For the global CCI values, variance was significantly higher in the young-old compared to the middle-old subgroup in the eyes-open hard and eyes-open foam conditions (*p* < 0.05), and in the middle-old compared to the old-old subgroup in the eyes-open foam condition (*p* < 0.05). There were no significant differences in variability between the young-old and old-old subgroups.

There were no significant differences in variability in lower leg CCI values between old-age subgroups.

### Age and condition-based differences in COP features

**Fig. 7:**
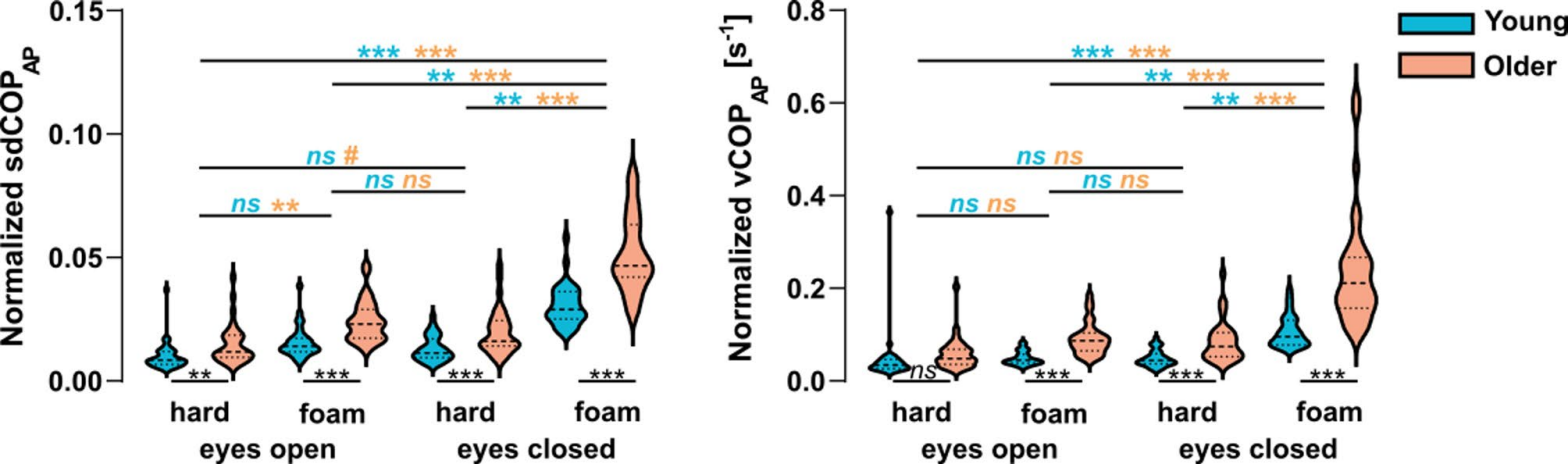
Distribution of postural instability parameters across conditions and age groups. Left depicts violin plots for sdCOP_AP_ and right for vCOP_AP_. All is as in Fig. 5. Significant differences between groups and between conditions are indicated with horizontal lines (ns, not significant; #0.05 < *p* < 0.1; **p < 0.01; ***p < 0.001).

Figure 7 presents the sdCOP_AP_ and vCOP_AP_ values for both groups in the 4 conditions. A two-way ANOVA applied to sdCOP_AP_ and vCOP_AP_ (separately) revealed a significant effect of condition (sdCOP_AP_, *F*_3,252_ = 40.9, *p* < 0.0001, η_p_^2^ = 0.33, 95% CI [0.23, 0.40]; vCOP_AP_, *F*_3,252_ = 36.6, *p* < 0.0001, η_p_^2^ = 0.30, 95% CI [0.21, 0.38]) and age (sdCOP_AP_, *F*_1,252_ = 23.8, *p* < 0.0001, η_p_^2^ = 0.09, 95% CI [0.03, 0.15]; vCOP_AP_, *F*_1,252_ = 15.8, *p* < 0.0001, η_p_^2^ = 0.06, 95% CI [0.01, 0.12]), and a significant interaction thereof (sdCOP_AP_, *F*_3,252_ = 4.4, *p* = 0.005, η_p_^2^ = 0.05, 95% CI [0.01, 0.10]; vCOP_AP_, *F*_3,252_ = 3.4, *p* < 0.02, η_p_^2^ = 0.04, 95% CI [0.00, 0.09]). Figure 7 presents the outcome of post-hoc comparisons. Overall, participants were increasingly more unstable with condition complexity, and the older participants displayed higher instability compared with the younger participants, especially in the most challenging condition.

### Behavioral correlations between CCI and postural parameters

Due to instability and fall risk being especially relevant for the elderly, we assessed the relationship between CCI values and postural instability within the older group. Moreover, to reduce the number of comparisons in the correlational analyses, only the condition that elicited the greatest instability, the eyes-closed foam condition, was analyzed. We identified high correlation between the instability parameters in the older group, sdCOP_AP_ and vCOP_AP_ (*r* > 0.80). This strong correlation suggests sdCOP_AP_ and vCOP_AP_ reflect a common component of postural instability, and justifies our decision to pool them into a composite instability measure.

The correlation analysis revealed significant relationships between global and knee extensor CCI values and pooled postural instability (*r* = 0.38 and *r* = 0.44, respectively; 0.01 < *p* < 0.04), where greater co-contraction was associated with increased instability. No significant relationship was identified for the lower leg CCI values (*r* = 0.23, *p* = 0.20). Controlling for age with partial correlation led to reduced association strength, where the global CCI and instability comparison was no longer statistically significant (*r* = 0.26, *p* = 0.16). However, the significant relationship remained for the knee extensor CCI and instability comparison (*r* = 0.37, *p* = 0.04).

**Fig. 8:**
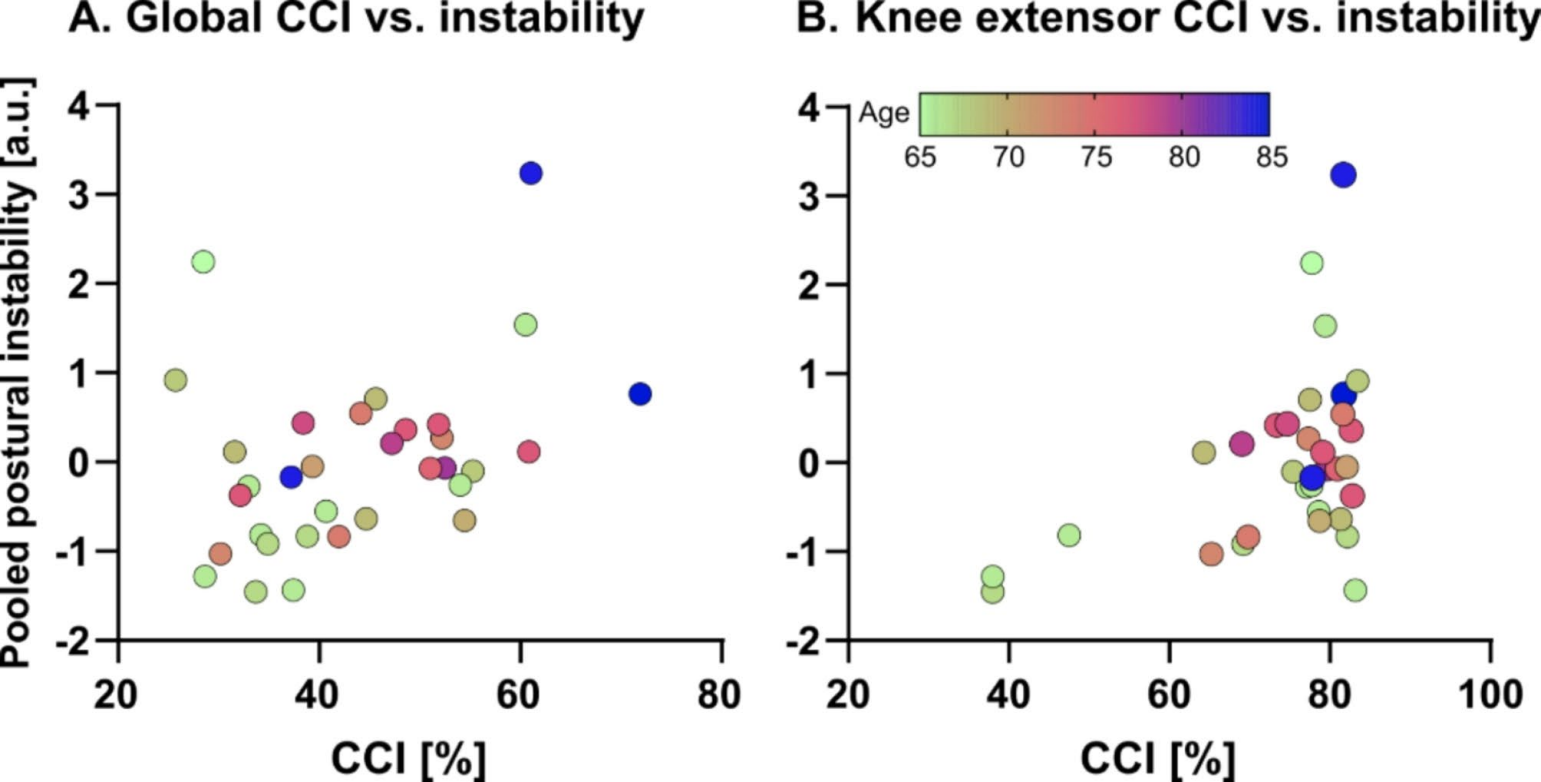
Association between global (**A**) and knee extensor (**B**) CCI values and instability in the eyes-closed foam condition. Circles indicate individual values and colors indicate age in years, according to the colorbar (bottom).

Figure 8 illustrates the association between global and knee extensor CCI values and pooled postural instability in the eyes-closed foam condition.

## Discussion

This study investigated the impact of aging on whole-body muscle co-contraction and lower-limb muscle synergies during quiet standing. We found that older individuals are more prone to using a knee extensors synergy, and displayed higher VAF. Even more striking, not only are older people co-contracting to a greater extent, but our fine-grained analysis revealed that there are distinct changes to the magnitude and variability of co-contraction with advancing age and postural challenge. More precisely, a high degree of co-contraction was seen systematically in the oldest individuals. Notably, condition complexity shifted the age threshold at which this co-contraction was seen systematically, where challenging conditions lowered the age at which such co-contraction was evident. Finally, we have identified significant relationships between co-contraction and postural instability, both at the global and local muscle level. These relationships were partly, but not fully, mediated by age, demonstrating the behavioral relevance of co-contraction for balance maintenance.

### Altered neuromuscular control of posture in aging

Older participants displayed increased co-contraction in muscle pairs within and across hemibodies, in muscle pairs spanning the extremities or trunk, and especially in muscle pairs containing the VL or VM. This latter finding was further supported by our muscle synergy analysis showing that the older group used a synergy containing the VL and VM more frequently than the younger group, which is also in agreement with previous work assessing muscle activations during walking^13,41^. Thus, the increased use of this muscle synergy and increased knee extensor co-contraction intensity across standing and walking tasks may suggest the existence of a common mechanism of altered motor control in aging.

Previous work argues that increased co-contraction in aging could be in response to altered mechanical properties of the musculotendinous unit and/or as a result of central nervous system changes. To overcome losses of muscle force or joint laxity, increased co-contraction may be necessary^42^. Other work points towards age-related changes to the spinal and cortical control of posture, typically with regard to changes in coactivation between agonist–antagonist muscle pairs^43^. Importantly, our results show increased coactivation within the same functional group, as well as between functionally opposite muscles. This pattern of co-activation could be explained by spinally mediated changes where decreased spinal inhibition within the knee flexor and extensor circuit drives extensors to become synergistically more active. Besides, some cortically mediated changes are also possible, where inaccuracies in the scaling of flexion, extension, and coactivation motor commands generate increased knee extensor activation (for a review, see^44^).

In line with altered central control of posture, we identified differences in VAF between age groups, where the older group had increased VAF across conditions. Previous studies have interpreted VAF as an index of the complexity of motor control, where higher VAF may represent decreased complexity^45–47^. The increased VAF in older individuals may suggest a reduced motor repertoire, constraining their ability to respond to postural perturbations, thus increasing instability. However, this is speculative^46^. Indeed, the potential mechanisms that give rise to a ‘simpler’ motor repertoire in older adults are unclear. Still, the aforementioned central changes may play a role.

When viewing our results with respect to neurological conditions, one may draw many parallels. Increased muscle co-contraction has been identified in several cerebellar ataxia types during walking^48^, as well as in Parkinson’s disease^49^, and cerebral palsy^50^. While our data cannot specifically comment on altered central physiology, the ubiquity of increased co-contractions in these conditions and in aging strongly suggests that central compensation is occurring. Whether the global muscle co-contraction strategy is a result of altered afferent processing^51^, physiological aging of the cerebellum^52^, extrapyramidal aging^53^, or other neurological changes should be the topics of future investigations. It is important to note that our study was cross-sectional, and therefore limits our ability to determine causality. While we interpret our results as rationale for central compensation, longitudinal and interventional studies are needed to clarify the mechanism at hand.

Aside from the knee-extensor synergy, two other synergies showed a differential use between groups, this time being more frequently used in the young group. The younger group used more frequently a synergy that was dominated by the activation of the ES bilaterally, as similarly identified in a gait-based experiment^12^. This could be indicative of a postural strategy where the young maintain a more upright (extended) spine and/or stabilize the pelvis by increasing the activity of these muscles and hence their synchronous activation^54^. Another report also demonstrated age-related differences in spinal and trunk muscles, but changes were dependent on anatomical location (thoracic, lumbar, etc.^55^). Thus, characterization of additional trunk muscles may call attention to a more nuanced control of spinal muscles, not captured by ES muscles only.

Although the muscle synergy analysis identified more frequent use of the ES-ES muscle pair in the young participants, the co-contraction analysis did result in age-related differences within this muscle pair. This is in contrast to other muscle pairs, that contained either ES muscle, that were significantly more co-contracted in the older participants. Thus, different information is contained within the synergy and co-contraction results. Broadly, synergy analyses enable the investigation of the modular control of movement, providing insight into the composition and frequency of use of particular groupings of muscle, while muscle co-contraction analyses examine how individual muscles interact within or outside of these modules. Together, they provide complementary information into the hierarchical control of movement.

Finally, the young used a right BF synergy more often. Such a synergy, featuring only one muscle, is not exactly a synergy in the physiological sense. Its existence in our data may indicate that this muscle, aside from co-contracting with the VL and VM, tends to activate independently of other muscles. The more frequent use of this synergy in young participants could complement the ES synergy in stabilizing the pelvis and/or serve to stabilize the knee joint, which has been suggested to contribute to successful functional movements^56^. Why this was significant unilaterally is difficult to explain, although limb dominance is a potential explanation. Future study of muscles located above the knee appear necessary in age-related postural work to clarify how these changes manifest and uniquely contribute to postural control.

### Contribution of co-contraction to (in)stability in old age

Increased co-contraction appeared to be the default strategy for maintaining posture in old age, as evidenced by elevated global co-contraction across conditions, and in combination with reduced variance in co-contraction among the oldest subgroup. This was made incredibly clear in the combined investigation of the mean and variability of knee extensor CCI values across the old age subgroups. With advancing age, older individuals tended to consistently co-contract with high intensity. However, the age threshold at which such co-contraction is systematic appeared to depend on the complexity of the condition. In the most challenging condition (eyes-closed foam), this occurred at age ~ 70, in the intermediate conditions (eyes-closed hard and eyes-open foam), at age ~ 80, and this did not occur during normal (unperturbed) standing, potentially due to the limited age range in this study.

Such co-contraction was not only related to advanced aging, but also postural instability. In examining the most challenging standing condition, we identified a significant relationship in older individuals: as both global and knee extensor CCI values increased, so did instability. That is to say, individuals co-contracting with greater intensity had more difficulty in maintaining stable posture. Importantly, this behavioral relationship between knee extensor CCI and instability was not fully mediated by age, suggesting that coactivation of the knee extensors may be a suitable index to assess mechanisms underlying instability, regardless of age.

Co-contraction in aging could be a compensation mechanism for age-related changes in joint mechanics, result from degraded central motor control, or even be the consequence of a fear-driven stability limit response^57,58^. In any case, such co-contraction is at best not fully effective in producing stable balance, this according to its association with instability in our cohort of older individuals. Another contributor to instability could be related to our finding of increased co-contraction of forearm muscles together with most of the other recorded muscles in older individuals, especially in the eyes-closed conditions. Indeed, upper limb movements generate reactive forces that can support and speed up balance correction upon perturbation. Forearm co-contraction and resultant arm restriction has been reported to impact static balance more in older compared to young individuals^59^. Other studies have also identified significant relationships between muscle co-contraction and instability^60^, supporting the notion that this motor strategy could be counterproductive. Though one may argue that this relationship only existed during substantial sensory perturbation and therefore lacks ecological validity, older individuals navigating their environment in the evening/night could very well experience decreased vision and altered standing surfaces (carpeted flooring, wood flooring, etc.), giving rise to similarly altered somatosensory information.

To speculate, it is possible that age-related increased co-contraction may complicate postural correction. When encountering an obstacle or perturbation, an individual who depends on a pure co-contraction strategy, which interrupts the typical motor control of standing and gait patterns^41^, would need to initiate another motor command requiring a separate timing and activation of the already co-contracted muscles in order to maintain an upright posture and prevent a fall^61^. This breakdown of the ongoing co-contraction motor command could contribute to delayed or inaccurate postural corrections, thereby increasing instability and potentially fall risk. Indeed, our results and this interpretation are in line with the theory of posturo-kinetic capacity, in which any factor (i.e. muscle co-contraction) that alters the dynamic mobility of the postural chain, would negatively impact postural adjustments, and would therefore alter postural stability^62,63^. Furthermore, while not studied here, increased co-contractions could accelerate muscle fatigue in the elderly due to higher metabolic costs and contribute to fall risk^23,61,64–66^.

Taken together with the fact that increased fatigue impairs balance and functional performance^67^, strategies to decrease co-contraction intensity in the elderly may improve functional outcomes. Indeed, our data indicate that co-contraction within the knee extensor muscle group is increased in older adults, evolves in advancing age, and is behaviorally disadvantageous. This suggests that novel interventions aimed to improve balance may benefit by targeting the knee extensor muscles. The question remains on the cause of this strategy and how to mitigate its associated instability.

### Limitations and perspectives

It is possible that differences in adiposity between groups could influence EMG-related results^68^. However, it should be noted that an almost identical cohort of young and older participants showed no significant differences in subcutaneous adipose thickness above the thigh or upper arm^69^, making it unlikely that adiposity influenced our results. On the other hand, muscle weakness is thought to play a role in mediating the age-related increase in co-contraction^70^. While muscle strength was not measured in this study, our previous work, again with the similar cohort of participants, identified significant differences in muscle size^69^. Thus, we cannot exclude this characteristic as a potential contributor to altered co-contraction in aging.

Additionally, some methodological choices, and in particular, the type of EMG normalization applied, may have influenced our results. Indeed, we elected to use a normalization by mean amplitude, thereby focusing on relative rather than absolute activation amplitudes. Still, future studies may benefit from complementary normalization strategies (e.g., maximal voluntary contractions or submaximal reference tasks). In our study, we calculated CCI values across all possible muscle-pairs (15 muscles on each hemibody), whereas previous CCI research estimated co-contraction between agonist and antagonist muscle-pairs. While these latter comparisons are more directly related to control of a singular joint, our analysis had the potential to highlight muscle-pair relationships that are typically unexplored, e.g. increased co-contraction between the VL and ES. Given our findings, this approach lends support to a central compensation mechanism which results in a diffuse spread of a motor command across disparate muscles. Other neuromuscular analytical approaches, like that of intermuscular coherence analysis, may be used in the future to determine whether stronger correlated neural input is evident not only within the lower leg, but across body segments. Already, recent work substantiates this idea, where older adults had stronger synchronization in low frequencies of posterior postural muscles compared to younger, potentially contributing to increased postural instability^71^.

Furthermore, the results of the old-age subgroups should be interpreted cautiously due to the limited subgroup sample size (n = 11). Future work should specifically investigate the evolution of neuromuscular postural control across the last decades of life and be powered to do so. In addition, a more comprehensive analysis including children and younger adults may further clarify other lifespan-related changes in neuromuscular postural control strategies.

Our work also included standing balance conditions that each lasted for 5 min, which is longer than typical postural control experiments and may be considered fatiguing. However, in a related analysis involving a similar cohort of young and older participants performing 5-min standing tasks, COP velocity assessed in consecutive 1 min intervals did not evolve over time^72^. This suggests that increasing task duration can only help improve the estimation of subject-specific postural stability, with little to no impact of fatigue.

Our results indicate that aging is accompanied with global muscle co-contraction and increased instability. Therefore, future work should determine whether interventions aimed at reducing co-contraction intensity may hold promise in mitigating instability. Such methods as the Alexander Technique^73^, which is a form of neuromuscular re-education designed to teach individuals how to improve postural support and reduce potentially harmful patterns of muscle tension may be useful. As well, stimulation techniques, either at the level of the cortex, or peripherally may be powerful tools to treat excessive muscle co-contraction, by means of decreasing motor cortical excitability^74^, or increasing inhibitory inter-neuron capacity, as is done to treat muscle spasticity in stroke^75^.

### Conclusion

Aging has clear effects on neuromuscular postural control strategy. The elderly tend to use a strategy in which they globally co-contract muscles across the extremities and trunk. Importantly, this strategy becomes increasingly common among individuals with advanced age and is associated with poor stability. Thus, global muscle co-contraction appears to be a hallmark of aging. Future work should determine whether it is a risk factor for falls that could be used as a potential diagnostic measure or target for interventions aimed at mitigating instability in the elderly. Critically, our findings underscore the importance of the inclusion of global muscle dynamics when studying postural control and lay the foundation for CCI analyses not restricted to agonist–antagonist muscle pairs.

## Data Availability

Data and analysis scripts used in this study will be made available upon reasonable request to the corresponding author.
